# Selective Plasma Etching of Polymeric Substrates for Advanced Applications

**DOI:** 10.3390/nano6060108

**Published:** 2016-06-07

**Authors:** Harinarayanan Puliyalil, Uroš Cvelbar

**Affiliations:** 1 Jožef Stefan International Postgraduate School, Jamova cesta 39, 1000 Ljubljana, Slovenia; hari.puliyalil@ijs.si; 2 Jožef Stefan Institute, Jamova cesta 39, 1000 Ljubljana, Slovenia

**Keywords:** plasma etching, selectivity, surface chemistry, nanomesh, nanostructuring, polymer composite, biomaterials, adhesion, etch mask, radical atoms

## Abstract

In today’s nanoworld, there is a strong need to manipulate and process materials on an atom-by-atom scale with new tools such as reactive plasma, which in some states enables high selectivity of interaction between plasma species and materials. These interactions first involve preferential interactions with precise bonds in materials and later cause etching. This typically occurs based on material stability, which leads to preferential etching of one material over other. This process is especially interesting for polymeric substrates with increasing complexity and a “zoo” of bonds, which are used in numerous applications. In this comprehensive summary, we encompass the complete selective etching of polymers and polymer matrix micro-/nanocomposites with plasma and unravel the mechanisms behind the scenes, which ultimately leads to the enhancement of surface properties and device performance.

## 1. Introduction

Plasma technology is one of the fastest developing branches of science, which is replacing numerous conventional wet-chemical methods in high-tech laboratories and industries, with a huge impact in renewable energy, environmental protection, biomedical applications, nanotechnology, microelectronics, and other fields. Plasma, the complex mixture of ions, radicals, electrons, and excited molecules has replaced conventional methods to develop various nanostructured materials with complex morphology and advanced properties (e.g., the production of vertically aligned carbon nanotubes (CNTs), which is difficult to achieve with other synthetic methods [1,2]). Owing to fast multi-scale production, it is a preferred method for the synthesis of other nanomaterials such as nanowires (NWs), carbon nanowalls (CNW), graphene sheets, *etc.* [3,4,5]. Through this, plasma in nanoscience also impacts the field of renewable energy and environmental protection, where we endeavor to replace hydrocarbon energy resources with solar energy or hydrogen fuel cells [6,7]. An additional benefit of plasma technology is the wet free doping of semi-conducting nanomaterials with heteroatoms to alter the band gap energy and conductivity for various applications [8,9,10]. In the overview of the applications in biology and medicine, plasma is found in various applications, from plasma surgery and the manufacture of artificial implants to the straightforward disinfection of the medical equipment.

The main processing routes in plasma technology are simply concluded into deposition and etching. For achieving the best material performance, these two techniques are either used individually or in a combination. Plasma-enhanced chemical vapor deposition (PECVD) is a technique complementary to thermally excited chemical vapor deposition (CVD) and is used for creating thin layer coatings, micro- or nanostructures, and even the deposition of complex functional materials [11]. Various applications of plasma deposition techniques are found in numerous articles, and some examples are given in [12,13,14,15]. On the other hand, plasma etching originates from the interactions between plasma particles with various substrates. The interactions are either physical or chemical; the particles with high kinetic energy are utilized to knock out the atomic or molecular species from the surface, whereas in the latter case the interaction of particles is purely potential. The physical interactions are mostly linked to ionized plasma species, while potential is linked to neutral or excited species.

Plasma etching, referred many times as plasma chemical etching or dry etching, of both organic and inorganic materials was reported for material fabrication in multidisciplinary applications. Compared to the wet chemical etching, plasma etching is capable of controlled and precise etching at very small scales (~10 nm). Additionally, plasma etching limits the disadvantages, for example, via contamination or solvent absorption during the treatment process. Some of the major advantages and disadvantageous of wet chemical etching *versus* plasma etching are listed in Table 1. In this review, we will mostly focus on the plasma etching of organic materials, which typically undergo etching relatively faster than inorganic materials. The difference in the etching arises from the stability of materials towards various chemical species. As an example, when we compare with liquid chemistry, alkali metals react explosively with water or acids, whereas transition or inner transition metals react very slowly or even stay inert. In the same way, different organic or inorganic materials react at different rates with various plasma species. This advantage of the disparate reactivity of substrates towards plasma is then utilized for fabricating important micro-/nanostructured materials.

In the field of material fabrication, the organic materials, especially polymer materials, are replacing many inorganic substrates. Simple fabrication and low production costs are the two great advantages for employing polymer materials. However, to minimize certain disadvantages of pure polymers, their properties are improved by making composites from multiple organic materials using multiplication of material properties or even reducing their dimensions to the nanoscale. To achieve this, the preferential removal of materials is desired, which is simply achieved by plasma etching. This approach was found to be the best method for many applications to achieve desired surface structures, morphology, or chemistry [16,17]. One should stress the effects on the surface chemistry, especially when it comes to organic materials, as it is typically more important than the morphology for certain applications such as adhesion. Plasma etching always alters the surface energy by the successful incorporation or formation of chemical groups during the interaction of plasma particles with materials [18].

**Figure 1:**
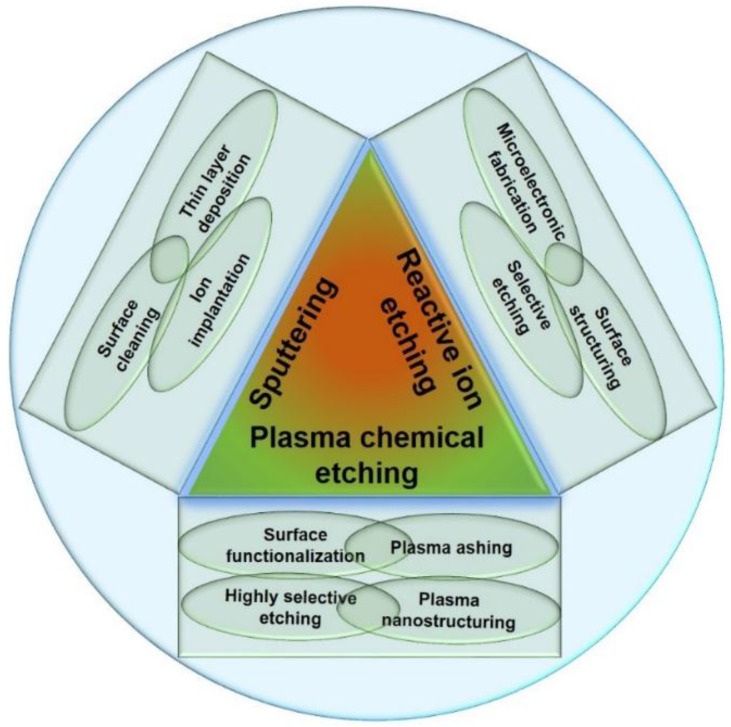
Schematic representation of various uses of different plasma processes.

Up to now, no reviews have reported specifically on the topic of plasma selective etching, which has innumerable applications in the field of materials science. This review concentrates on the applications of selective etching. However, prior to that, a short discussion is made on the important types of plasmas based on particle species, their energy, and the result of interaction with the surface, namely sputtering, reactive ion etching, or neutral radical chemical etching. All these interactions then drive the representative applications of plasma etching schematically presented in Figure 1. Sputtering, which involves the removal of various substrates merely by high-energy particle collision is not very important for the selective etching of materials. On the contrary, the reactive ion etching and the neutral plasma chemical etching are found to be efficient for the preferential removal of one material over the other. Following this, the applications of the plasma modified polymeric substrates are described. The main objective of this article is to focus on the characteristics of polymer materials, which are decisive for the etching rate and stability towards high-energy particles. Plasma processing of various polymer composites and their advanced applications are outlined as well.

## 2. Plasma Processing

### 2.1. Sputtering

Sputtering deposition is widely employed as a robust method in a thin film preparation for countless applications. The basic principle of the sputtering technique is the generation of high-energy ions, which are then accelerated and guided towards the substrate where they knock out surface atoms as ions into the vapor phase. The so-generated ions are also guided and deposited on the coated substrate where they form a thin layer. However, this deposition process is beyond the scope of this paper. More interesting are energetic surface collisions, where material is removed from the surface.

**Figure 2:**
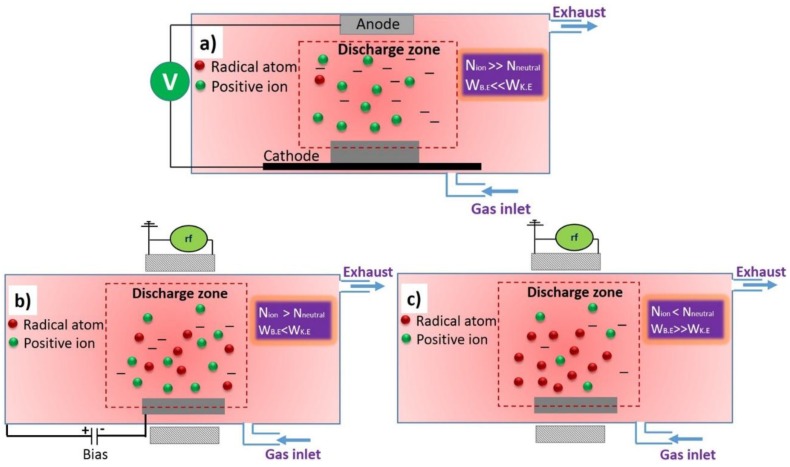
Schematic representation of various plasma processing systems for (**a**) sputtering; (**b**) reactive ion etching; and (**c**) highly dissociated weakly ionized plasma for chemical etching.

While considering the process, one should create an ion-rich environment that enables the acceleration of generated particles by applying an external electric or magnetic field. The number density and kinetic energy of the ions (N_ion_ and W_K.E._, respectively) should be sufficiently large to overcome the energy barrier created by the binding energy of the surface atoms (W_B.E._). The sputtering process is efficient when the kinetic interactions significantly prevail over chemical and thermal interactions, which typically occurs at ion energies ranging higher than several 100 eV or even higher than 1 keV [19]. However, the sputtering process efficiency in atom removal is significantly dependent upon the material sputtered. One simple schematic representation of the plasma-sputtering chamber is presented in Figure 2a.

The major drawback of the sputtering process is its inefficiency for the surface modification of delicate materials including polymers. The problem is that the high-energy particle–surface interactions are uncontrollable and completely damage the surface mostly through cascade collisions inside the material, which provide almost no selectivity. Moving away from high-energy ions, which cause only the pure kinetic-physical interactions of ions with surfaces (via two body collisions), there is a gray zone of so-called chemical sputtering, where some chemical reactions between the incident beam and substrate occur. This process involves mostly lower energy ions (typically with energies around 100 eV) and is commonly applied to remove the surface contaminates such as hydrocarbons [20].

### 2.2. Reactive Ion Etching

Reactive ion etching (RIE) was mostly developed and improved by the microelectronic industry in the last few decades for trenching and patterning the surfaces. The RIE process is very diverse and uses various plasma combinations, with species ranging from merely low-energy ions (10 eV to several 100 eV) to combinations of ions with radicals, excited atoms, and electrons. These combinations are mostly generated with glow discharges, where the ratio of (W_K.E._) to (W_B.E._) is much lower than in the case of sputtering (Figure 2b). In these processes, the etching material is typically DC-biased in order to increase the effect of ion collection and acceleration towards the surface. The process is most commonly done inside a chamber with low pressure of a selected gas, where plasma is generated in a high-frequency discharge [21]. In such systems, where the surface potential is controlled, the interacting ions are directed, their energy distribution controlled by bias voltage. Nevertheless, other reactive species including neutrals inside the plasma play a significant role in determining the etching rate and etching anisotropy. Like in the case of any plasma process, the etching rate inside a reactive ion chamber is partially dependent on the discharge parameters that influence plasma properties. Therefore, the increase in the discharge power improves the etching rate, since the dissociation efficiency increases with the applied power. On the other hand, the generation of self-bias potential at reduced pressure improves the etching efficiency by energizing the particles [22].

The most reported discharge systems used for RIE are capacitively coupled RF plasma (CCP) and inductively coupled RF plasma (ICP). In CCP plasma, the ion energy varies as a function of applied power, whereas an increase in gas pressure reduces the acceleration of ions by collisions with the background gas. The ion energy flux to the surface is increased by bias voltage, which imparts a high kinetic energy to the particles. This makes complications by reducing the control or selectivity over the etching process. However, lowering the pressure also results in an increase in the collision mean free path, which restricts the existence of plasma at the point in which the mean free path approaches a value that is of the same order than the distance between the electrodes [22,23]. On the other hand, ICP is capable of generating high particle densities without any additional biasing, even at higher gas pressures [24].

The etching rate in RIE processing varies from a few nm to a few hundred nm per minute depending on the conditions used for the processing [25]. Optimization of the process is extremely important because of the problems encountered during RIE such as bowling, undercutting, or mask scattering, all of which adversely affect the aspect ratio of the processing, especially during the fabrication of circuit boards [26]. To improve the etching rate and aspect ratio, RIE is sometimes combined with other processing techniques including magnetic-field-induced beam control, lithography, ultraviolet (UV) irradiation, *etc.* [27,28].

### 2.3. Neutral Radical Plasma Chemical Etching

Very frequently, processes like ion sputtering and even RIE lead to thermal damages of the surface or even of bulk properties, which make them improper for the etching of temperature-sensitive materials such as polymers. In order to avoid overheating by ion bombardment or even by increased neutral gas temperature, it is wise to use mostly neutral plasma species like neutral atoms. This is achieved with so-called cold plasmas mostly generated at lower pressures (around 100 Pa) with high-frequency discharges. The high frequency applied accelerates the electrons, whereas the heavier ions cannot follow frequency, which results in a high dissociation and low ionization of the feeding gas (Figure 2c). When treatments are performed in the post-glow or after-glow regions, the discharge features practically no ions. Microwave discharges are popular for the plasma treatment of materials because they are characterized by low plasma potential, which has the advantage of insignificant ionic effects, but the neutral gas temperature is normally high. On the other hand, RF discharges have the electron temperature of about 5 eV for plasma gases such as O_2_, where the observed neutral gas temperature is lower compared to microwave discharge. In such high frequency discharges, the dissociation is almost linearly enlarged with the increase in discharge power, independent of the gas flow [29].

Highly dissociated plasma is not only applicable for polymer etching, but also for other applications such as chemical reduction, nanostructuring, and plasma cleaning [30,31,32]. The reactions of polymers and other materials will be more elucidated in the following chapters, where different neutral atoms contribute a major part to etching through adsorption and recombination processes [33,34,35]. The recombination of atoms at preferential spots on the surface originates from the roughness of the surface and plays a crucial role, even in deciding the nanostructure growth [24,36]. The surface interactions and bond-breaking mechanisms during the interactions of non-equilibrium plasmas with various carbonaceous materials are also reported [37,38].

## 3. Applications of Plasma Functionalization and Etching of Polymers

Polymer materials cover a large segment of our material requirements for packaging, microelectronics, photonic devices, medical implants, sealing applications, water repellent coatings, thermal and electrical insulators, sensing materials, *etc.* [39,40,41,42]. Plasma-assisted surface functionalization and etching were proven to provide the desired surface energy and morphological changes to the polymeric surface [43,44]. Although various chemical treatments such as soaking in acidic medium are efficient and inexpensive methods for improving the surface energy of polymeric materials, they are associated with persisting residuals after treatment and, to a large extent, connected to environmental pollution [45,46]. Plasma modification consists of surface functionalization, which is considered to be a primary step in which surface chemistry is altered by bond breaking and the incorporation of functional groups. Thereafter, the removal of material takes place through the bonding of surface atoms with impinging radicals, which recombine and leave the surface. Through this mechanism, the organic surface contaminants or weakly bonded surface layer are removed. An alternative to plasma pre-treatment is UV irradiation, which mostly results in surface functionalization, but it is not sufficient for material etching. Moreover, wet chemical treatments as the second alternative are limited by the type of incorporated functional groups [47]. By taking these factors into consideration, plasma treatments are preferably used in polymer treatments for a wide variety of applications.

Among various applications of plasma surface treatments, improving the adhesion of various metals on polymer substrates is an important task. The major challenge in metallizing polymeric surfaces is their lower surface energy, which confronts the adhesion of metallic particles. By incorporating suitable polar functional groups by plasma exposure, this deficiency is easily solved. For instance, Pascu *et al.* presented both microwave and RF plasma treatments of polyvinylidene fluoride with N_2_ and NH_3_ as working gases for the incorporation of polar nitrogen containing groups including amines, nitriles, or imines. These incorporated polar functionalities then aided to increase the amount and adhesive strength of the deposited copper [48]. Metallized polymers including polyethylene terephthalate (PET) are important in the fabrication of microelectronic and photonic devices, where the intermixing of the metal and polymer showed the significant improvement of their properties when material was exposed to post-deposition annealing in Ar or Ar/O_2_ plasma [49]. The treatment of the samples was done with Ar/O_2_ plasma generated at low pressures (50–100 Pa) within RF discharge at power 35 W. The process increased the surface roughness and porosity, allowing the diffusion of coated Au/Ag metal from the surface into the bulk. To obtain the best adhesion, the plasma treatment conditions were optimized to minimize fracture at the interface between the metal film and polymer substrate [50,51]. The adhesion of Cu layer on polyamide also increased with O_2_ plasma treatment at 200 W and 8 Pa for 3 min prior to Cu metallization. The peel strength of the deposited Cu on polyamide increased to a value of 250 g/mm, which was almost 280 times higher than that of the non-treated sample [50]. Irrespective of the gas or discharge condition used for the treatment, the metal coatings of polymers should be done immediately after the plasma surface modification in order to eradicate the drawbacks arising from the aging effects [52,53].

In addition to the improved adhesion of inorganic particles, plasma treatments have a significant role in fabricating surfaces, which are customized for the adhesion of various organic or biomolecules. Surface treatment with oxidizing plasma source gases such as O_2_, NH_3_, N_2_, Air, or even noble gas mixtures is found efficient to enhance the surface energy and thereby adhesion of various bio materials onto the surface [54,55]. The surface roughness and polarity provided by plasma exposure are utilized to increase the adsorption of anti-blood clotting agents such as heparin onto various polymeric substrates. This operation is extended in application for manufacturing artificial organs [56]. In addition to this, the cell growth on various polymeric surfaces efficiently increases after suitable plasma pre-treatments as a simultaneous effect of improved surface contact area and the electron rich behavior. The incorporated functional groups allow for the nucleation and adsorption of desired biomaterials on the surface of the implant used by which the spatial alignment of the bio-molecules on the surface is regulated [57,58]. Another supportive application of plasma etching in the bio-medical field is plasma sterilization of surgical devices, drug packaging, or processing of other medical objects [59,60]. For example, on exposure to reactive O_2_ plasma neutral radical in the afterglow, the blood proteins and microorganisms showed exponential etching rate, whereas the PET substrate exhibited only a linear etching rate [61]. These distinct etching rates help to remove toxins or other organic contaminants from the surface without affecting the surgical equipment or medical material. Further applications of plasma degradation of materials extend into novel applications such as decontamination of toxic warfare agents, the cleaning of dental devices, and even the precise removal of cancerous cells [62,63,64].

**Figure 3:**
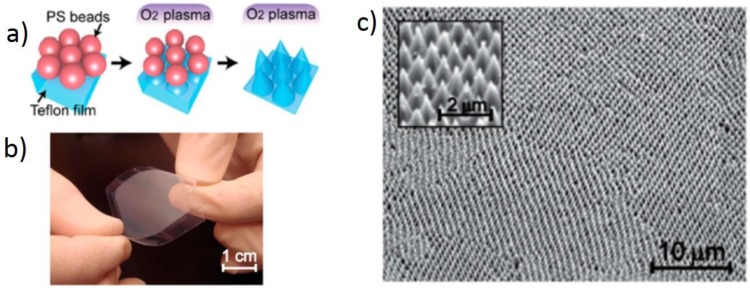
(**a**) Scheme of the fabrication process of Teflon nanocone arrays; (**b**) Photograph showing a macroscopic view of flexible Teflon nanocone array; (**c**) Scanning electron microscopy (SEM) images of the tilted nanocone array. Inset: detailed view of Teflon nanocones (Reproduced with permission from [69]. Copyright American Chemical Society, 2014).

Besides the improved surface energy of polymeric substrates, plasma treatment is also able to incorporate low-energy functionalities on the polymeric surface. This is largely applied in the fabrication of water-repellent surfaces, which are significantly important in terms of generating better anti-aging or anti-corrosive properties of membranes for oil-water separation, and of fluid transportation control [65,66]. The self-cleaning ability of materials is expressed in terms of the water contact angle, where the surface with a contact angle above 150° is termed as the superhydrophobic surface [67]. Plasma etching efficiently creates superhydrophobic surfaces by two major mechanisms: firstly by providing sufficient roughness to the surface by the etching process and secondly by providing a sufficient number of low-energy functional groups [68]. Typically used fluorine containing plasmas have an ability to simultaneously incorporate sufficient amounts of low-energy functional groups as well as create the desired surface roughness through etching. In the cases of fluorinated polymers including Teflon (polytetrafluoroethylene—PTFE), even fluorine-free gases are sufficient enough to generate hydrophobicity merely by increasing the surface roughness by polymer etching [66,69]. An example of this are nanocone structures on Teflon surface achieved after etching the surface with O_2_ plasma (25 Pa and 50 W) for 10 min. In this case, the water contact angle achieved was 134°. Such nano-featured surface is achieved also by etching anisotropy, which is created with deposits like polymer beads. The surface decorated with polystyrene (PS) beads with a 10-µm diameter prior to the plasma process can create nanocones during etching as well (Figure 3) [69]. The sufficient roughness is boosted by the additional deposition of Au nanoparticles on top of the nanocones, which leads to a superhydrophobic surface response. In certain cases, the roughness on the etched surface is influenced by the sputtered deposits from the plasma chamber wall, which act as a mask to protect the polymer but provide similar anisotropy for etching [70].

## 4. The Origin of Plasma Etching Selectivity

The importance of the dry etching of polymeric materials for numerous applications such as masking or nanostructuring has increased the demand for systematic studies on the stability of various polymeric materials towards reactive plasma particles. The stability of the polymer definitely depends on the type of polymer/monomer units present (homo or copolymer, aliphatic or aromatic, crystalline or amorphous, *etc.*) as well as the energy and type of plasma particle interacting with it. Herein, the important factors are directly related to the stability of polymers towards plasma particles, which result in selective etching that will be presented and discussed.

**Figure 4:**
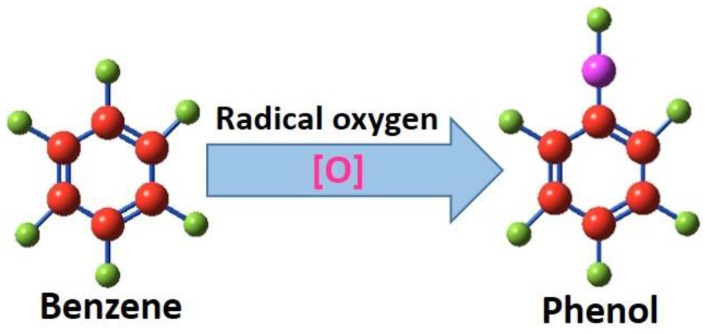
The schematic of reaction involved in the radical quenching by the aromatic ring to form functional group instead of ring cleavage.

One of the primary structural features that are considered is the aliphatic and aromatic moiety on the polymer backbone. The etching rates for aromatic polymers are relatively lower compared to aliphatic ones due to the extra energy stabilization (~36 kcal/mole), provided by the aromaticity of the rings in the polymeric chain. One more explanation to support the distinct etching rate is that the aliphatic rings degrade easily to form volatile molecules, whereas the aromatics generally form more non-volatile fragments and are not easily removed from the surface [71]. However, plasma-initiated degradation can occur on the bond–ring junctions, which are connected through relatively unstable secondary or tertiary carbon atoms. This argument can question the extra stability of the aromatic polymer chains exposed to the plasma. An appropriate argument was given by Taylor and Wolf, who stated that the extra stability of the aromatic polymers, irrespective of the position of the aromatic ring, originated from the ability of aromatic rings to quench the reactive plasma particles [72]. The quenching is typically directed through the abstraction of the hydrogen from the aromatic ring by the incoming reactive atom and the formation of a functionalized ring (Figure 4). This reaction prevents the formation of any active sites or dangling bonds on the polymer chain and prevents the chain cleavage. Additionally, the energy released during this process will be readily reduced by contributing to the vibrational energy of the polymer backbone [73]. Some early reports on the gas phase reactions of aromatic rings with oxygen plasma radicals significantly support this argument [74,75]. During such reactions, the hydroxylation was mostly directed towards the ortho position regardless of the size of the substituents on the ring due to the higher degree of neucleophilicity of oxygen radicals. This observation points out that the reactions are mostly guided through a kinetic route to give rise to reaction products that are thermodynamically less stable [74].

**Figure 5:**
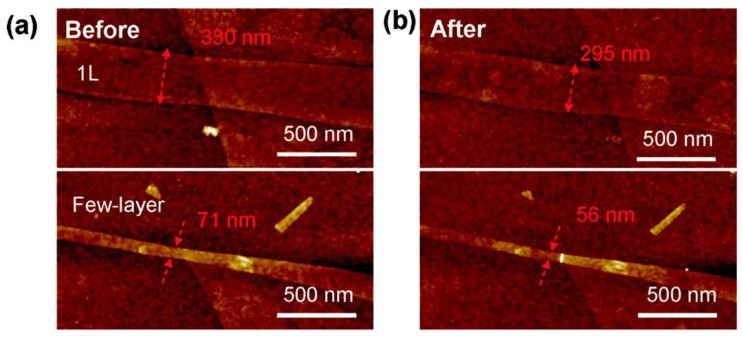
Atomic force microscopy (AFM) images of two small pieces of graphene (top: a monolayer (1 L) graphene strip; bottom: a few-layer graphene strip). (**a**) Before and (**b**) after selective hydrogen plasma edge etching for 60 min at 300 °C (Reproduced with permission from [76]. Copyright American Chemical Society, 2010).

Liming *et al.* provided an excellent example to present the etching efficiency and selectivity of plasma towards various chemical bonds [76]. The stability of various bonds was accurately distinguished by high-energy hydrogen radicals and ions, used to selectively etch away the edge atoms of graphene sheets and to convert them into graphene nanoribbons (GNR). As observed from atomic force microscopy (AFM) imaging, the observed etching rate for single layer graphene was 0.27 nm/min, whereas multi-layer graphene disclosed an etching rate of 0.1 nm/min (Figure 5). By keeping the surface temperature to an optimum value of 300 °C, the defect-free hydrogen terminated graphene nanoribbons were synthesized. The selectivity originated from the lower chemical stability of the edge carbon towards plasma particles compared to the atoms embedded in the mid-region of the graphene sheet [77]. In the same manner, while considering the polymeric substrates, regardless of the etching mechanism, the etching rate was mostly connected with the energy required for breaking the polymer backbone, as demonstrated by Taylor and Wolf. They compared the etching rate of different polymers (k_rel_) with the number of chain scissions per 100 eV of energy absorbed (G_s_) (Table 2). The etching rate displayed a linear relationship with the bond-breaking energy of the polymer backbone [72].

Another important parameter which determines the etching selectivity is the energy required for the initiation of chemical reaction (activation energy). For example, the chemical oxidation of Mg in air is highly exothermic. However, the reaction will not commence unless sufficient heat is provided such that the reactants can reach the intermediate transition state (activated complex). Once the reaction starts, the exothermic energy released will be used for the reaction to proceed. The comparison of activation energies required for the reaction between n-butane with various neutral species in the vapor state discloses that O (^1^D) and F atoms do not need any activation energy for the chemical reaction to start, which reduces the selectivity towards various hydrocarbon bonds. Although O (^3^P) species are reactive, the energy of activation is found to be 1 eV to react with the hydrocarbon, which indicates that this species can distinguish various chemical bonds much better than O (^1^D) or F species [73,78]. Likewise, the Cl atomic species are highly reactive without any bond selectivity towards the reacting polymer due to no activation energy barrier. On the other hand, Br atoms are relatively non-reactive due to an energy barrier of around 1.8 eV. Moreover, the etching rate can also be controlled through the combination of various reactive species, which can be regulated by controlling the feeding gas composition and discharge parameters. In one recent report, it was demonstrated that adding O_2_ into CF_4_ plasma increased the etching rate [79]. This phenomenon was due to the involvement of excited oxygen radicals in the electron impact dissociation of CF_4_, which leads to a higher dissociation and more F atomic species [80].

**Figure 6:**
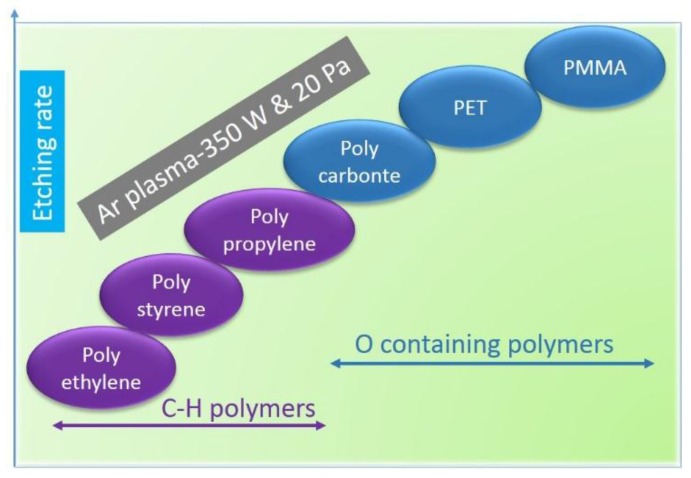
Etching rates for various polymer substrates in Ar plasma based on [81].

Hegemann *et al.* compared the etching rates for various polymers with different chemical structures inside the noble gas-generated plasmas and concluded that the polymer backbones that contained oxygen functionalities etched away relatively faster. For example, polymethyl methacrylate (PMMA), PET, *etc.* showed relatively higher etch rates compared to the hydrocarbon polymers inside Ar plasma generated at 300 W and a pressure of 20 Pa (Figure 6) [81]. The explanation for the higher etch rates for functionalized polymers was due to the secondary reactive oxygen atomic species released into the system as a result of polymer bond cleavage. More surprisingly, polypropylene (PP) exhibited a lower etching rate compared to PS, which was in contradiction to the hypothesis of radical quenching mechanism proposed for the extra stability of aromatic polymers. However, this was explained by the efficient cross-linking on the PP surface upon exposure to plasma due to the side chain activation [81].

**Figure 7:**
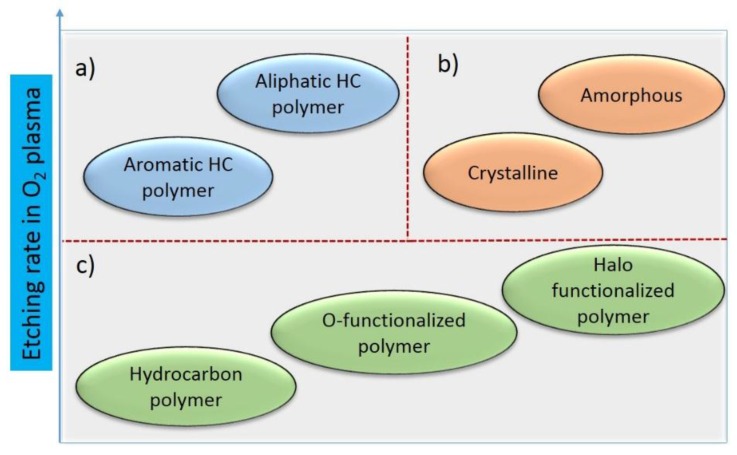
General schematic of the etching rate for various types of polymeric materials in O_2_ plasma. (**a**) Dependence of etching rate on the aliphatic/aromatic behavior of the monomer units; (**b**) Etching rate dependence on the crystallinity; (**c**) Functionality dependence of the polymer with etching rate.

Plasma surface interactions have a strong correlation with the crystallinity of the polymer used, where the etching rate is typically reduced with the increase in crystallinity [82]. The reason for this can be different neutral atom recombination probabilities on surfaces. For example, the neutral O atom recombination probability is 8.3×10^−4^–2.1×10^−4^ for amorphous PET or 4×10^−4^–2.5×10^−5^ for semi-crystalline PET [83]. Thus, the extension of interactions between the polymer and the neutral atomic species that induce etching differ with respect to crystallinity, which cannot hold for charged species. The ion flux to the surface is independent of the crystallinity. The increased value of the neutral atom recombination coefficient provided higher contribution to the surface temperature which induced more deformation to the amorphous material, as reflected by the roughness measurements before and after plasma exposure [83,84,85]. The treatment of LDPE (low density polyethylene) and HDPE (high density polyethylene) inside CF_4_ plasma revealed similar results. The etching rate for LDPE was 1.5 nm/min more than that of the HDPE inside CF_4_ plasma generated by capacitively coupled RF discharge, where the morphological changes also displayed significant differences with respect to crystallinity [86]. Nair *et al.* recently reported the removal of an amorphous carbon layer from the multiwalled carbon nanotube (MWCNT) with low-energy ions, radicals and metastable species, where the crystalline phase remained relatively intact. The extra stability provided by the crystallization energy additionally supported the etch-resistant behavior of crystalline polymers [87]. From all of the above-mentioned results, the relative etching rates for various polymeric substrates inside the widely used oxygen plasma are summarized in Figure 7.

An important class of polymers that needs to be separately addressed is that of block copolymers, since they are largely used in block copolymer lithography for the semiconductor industry. The chemical dissimilarities between the two polymer domains permit the selective etching of one component over the other, when a suitable treatment method is used. After the selective etching of the less stable polymer, the unaffected component will form the template pattern. One of the most widely used copolymers is the PS/PMMA system, where both of the components have distinct chemical stabilities. For achieving spherical and cylindrical structures, PMMA is easily removed with a suitable wet chemical treatment [88]. However, the wet chemical etching is limited due to capillary forces when the polymers possess lamellar arrangement. In order to avoid these problems, plasma selective etching is implemented and used for a number of copolymer systems: for example, polystyrene–poly ferrocenylisopropylmethylsilane diblock copolymer, styrene–butadiene–styrene (SBS) triblock copolymer, polystyrene–polydimethylsiloxane block copolymer, polyhydroxybutyrate-co-hydroxyvalerate, *etc.* [89,90,91]. The most fundamental object while choosing the copolymer for plasma etch patterning is the selection of proper blocks that have distinct etching rates towards various plasma particles [92].

The oxidation probability of the polymer is independent of the density of plasma particle species in the proximity of the samples, but the etching still depends on plasma particle properties and their fluxes to surface [93]. In many cases, the etch-resistant properties of polymer materials are dependent on the type of discharge used, where the difference in the etching rate originates from the energies and flux of various reactive species including neutral atoms and ions generated [37,94,95]. The etching rate is also well correlated to the surface temperature originated from the ion bombardment, neutral atom recombination, and exothermic carbon oxidation [96,97,98]. In order to achieve the highest etching rates, a higher surface temperature is always preferred. However, the surface temperatures above the glass transition temperature of the polymer can critically affect the bulk properties. Due to this, the radical and ion flux should be optimized, whereas in many cases the pulse mode plasma treatment over continuous mode will be more appropriate to control the temperature related effects [99,100,101].

## 5. Applications of Plasma Selective Etching of Composite Materials

Plasma selective etching is utilized in many applications for material fabrication especially in micro- and nano-structuring. One of the important examples is the transplantation of nanostructures like nanotubes. Li *et al.* demonstrated a method for growing CNTs inside Si trench with Ni catalysts. The challenge faced was the transfer of the designed nanotubes onto suitable receptors. This difficulty was solved by extracting the nanotubes with an epoxy matrix in the form of pellets, followed by oxygen plasma selective etching of the epoxy matrix in order to release well aligned and densely packed CNT arrays [102]. By taking advantage of the difference in etching selectivity of various chemical bonds towards plasma, the metal single-walled carbon nanotube (m-SWCNT) were also obtained. One of the challenges for creating m-SWCNTs is the higher chemical etching rate of m-SWCNTs compared to semi-conducting single-walled carbon nanotubes (s-SWCNT) [103]. However, by taking advantage of the weaker stability of smaller diameter s-SWCNT due to the C–C bond bending, a mild hydrogen plasma treatment yielded m-SWCNT from a mixture of m- and s-SWCNT [104]. This etching selectivity is only diameter-dependent, unlike the m- or s- character of SWCNT, and could yield a 100% recovery and easy scaling up of one type of SWCNT from the mixture [105].

Graphene-based electrodes are used in wide variety of semiconducting devices such as field effect transistors, sensors, supercapacitors, *etc.* [106]. In the transistor applications, one of the most important parameters is the gap dimension between the electrodes. Graphene sheets, which are separated by micro- or nanoscale distance, played a significant role in the fabrication of numerous electronic devices. The fabrication of such devices is extremely hard by wet chemical treatment due to the lack of precision in etching over nanoscale dimensions. As demonstrated by Liao *et al.*, plasma-assisted mask etching can provide precise control over the gap between graphene sheets ranging from a few micrometers to hundreds of nanometers [107]. Furthermore, the electrical properties of graphene sheets are highly dependent on their specific surface area. For this, graphene sheets can be converted into different porous forms including graphene nanomesh, crumpled graphene, folded graphene, and graphene foam in order to exploit the exceptionally large surface area [108,109,110]. Such high surface area graphene sheets are also easy to obtain by means of polymeric or metallic mask-assisted plasma selective etching. In a presented example, the spin coating of PMMA on graphene-supported silica, when covered with porous anodized aluminum oxide resulted in a suitably masked surface for plasma etching. Treatment of this composite with O_2_ plasma for 30 s resulted in pores with diameters of 67 nm on the graphene surface, which produces the graphene mesh with a very high surface area of 100 μm^2^ [111]. The origin of etching selectivity during the processing was based on the tolerance of the Al mesh towards plasma compared to the PMMA layer. The observed difference in the etching rates of PS and P(S-r-MMA-r-GMA) (a random copolymers of styrene, methyl methacrylate, and glycidyl methacrylate) was 1.17 and 1.42 nm/s, respectively, inside O_2_ plasma (50 W, 10 sccm, 1.3 Pa), compared to 0.76 and 0.96 nm/s, respectively, inside the CHF_3_/O_2_ gas mixture (300 W, 45 sccm CHF_3_ and O_2_ 5 sccm, 8 Pa). Exploiting this, the patterned copolymer template was fabricated, which provided an easy route for creating nanoperforations on graphene sheets. Such prepared graphene with a large surface area has been used in electronic and sensing devices [112]. Additionally, the combination of holography and photoresist-assisted O_2_ plasma selective etching was demonstrated for providing a low-cost production route to synthesize graphene nanomesh [113]. Additional examples are also seen where self-assembled masks are used for the perforation of graphene sheets during selective etching [114]. Such masks provide controlled exposure of the graphene layer to the plasma reactive species and yield precise mesh sizes and ribbon widths between successive meshes. This allows control over the electronic characteristics of graphene [115]. During the etching of such graphene composite surfaces, the selective edge oxidation and O_2_ physisorption on the inner walls of the mesh add secondary benefits to chemo-sensing applications [116].

Another particular application of selective plasma modification and etching was to improve gas permeation properties of composite membranes [117]. After plasma treatment with corona discharge, the polyamide 6/polyethersulfone (PA6/PES) composite membrane exhibited significant improvement in separation of CO_2_, N_2_, and O_2_ by incorporating functional groups and increasing the surface roughness [118]. Unfortunately, the changes induced to the distinct composite components were not studied individually. When a polybutadiene/polycarbonate (PB/PC) membrane was exposed to the Cl-containing plasma, it was observed that the modified surface chemistry had very little influence, whereas the separation towards N_2_ and O_2_ were dominantly affected by the variation in surface morphology [119]. Other than for permeation applications, selective plasma-assisted mask O_2_/Ar etching of Nafion membrane has been used in the preparation of patterned metal-polymer composite membranes for robotics and other applications [120].

Unzipping CNTs under plasma exposure is known to ease the preparation of novel materials including hollow CNTs or GNR. To make such preparative methods easier, the simplest method is to expose CNTs embedded in a polymer matrix to reactive plasmas. Exposure of the vertically aligned CNT in the polymer matrix to plasma opened the tips of the embedded CNTs. This route also included the removal of the polymer matrix at higher rates and then exposing the tube tips and further opening of the CNT tips [121]. In another relevant example, plasma selective etching enabled the successful synthesis of single-layer GNR from CNTs embedded in PMMA composite. The method provided excellent control over edge smoothness and uniformity in width for the fabricated ribbon structures [122].

Due to its simplicity, and the higher rate of dimension tolerance compared to wet chemical etching procedures, plasma etching is well exploited in the semiconducting industry for printing integrated circuit boards. One of the disadvantages often given for plasma processing is the high production cost due to expensive low-pressure plasma systems, which can be bridged only with long-term operation. Silicon wafers, as one of the most used materials in the semiconductor industry, is efficiently etched mostly by halogen-containing plasmas or by their mixture with other gases in the presence of a suitable etch mask. The etch mask on the surface generates an unbalanced etching rate of the surface and creates desired patterns on the wafer [123]. For achieving the etching selectivity between the semiconducting layer and the photoresist, the control of the plasma species inside is needed. By adding certain gases, it is possible to scavenge the radicals and ions, which presents an alternative route to controlling the species inside the plasma and the corresponding etching selectivity, without changing the discharge parameters [124]. One of the major drawbacks of RIE technology is the non-uniform etching on the sidewalls. It operates in such a way that the radicals get accumulated nearer to the edge of the wafer instead of the center, which increases the etch rate towards the edges. Additionally, the ion current is favorably drawn over the wafer edge, and the etch rates for the silicon wafer show an increase from the center to the edge and the consequent edge breakings [125]. Performance level and many of the disadvantages of RIE processing such as anti-notch performances, quality of the profile, surface finishing, *etc.* are tuned by adjusting the applied frequency or pulsing [125]. Even in the presence of these disadvantages, plasma etching is far better than other conventional methodologies.

**Figure 8:**
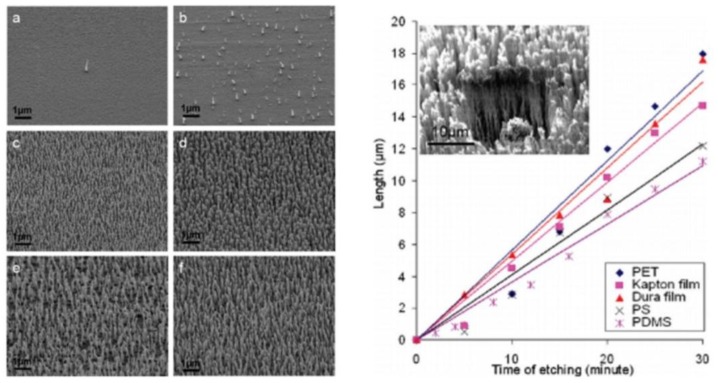
SEM images of the density-controlled fabrication of polymer nanowire (NW) arrays of Kapton by covering the initial surface with (**a**) 0.75; (**b**) 1.5; (**c**) 3; (**d**) 4.5; (**e**) 10; and (**f**) 15 nm of Au before inductively coupled plasma (ICP) etching. The graph represents the length-controlled growth of NWs of polyethylene terephthalate (PET), Kapton film, Durafilm, polystyrene (PS), and polydimethyl siloxane (PDMS). The inset is a SEM image of a NW array on Durafilm after 30 min of etching (Reproduced with permission from [128]. Copyright American Chemical Society, 2009).

An important application of the diverse material etching rates in plasma is used to deal with the nanostructuring of the polymeric substrates. Polymer-based 1D and 2D nanostructures have potential applications in sensing and energy devices [126,127]. Although template-assisted synthetic methods are well-known, controlling the dimensions during template removal still represents an obstacle. Plasma-assisted mask etching has proven to be an alternative to overcome these problems. An example is the exposure of metallic nanoparticles deposited on polymer surfaces to reactive gas plasma, which yields the dense polymeric NWs. The metallic coating or, say, metallic material islands (nanodots) merely acted as an etch mask to provide a rough surface to mobilize the NW growth [128]. For this purpose, a mixture of Ar, O_2_, and CF_4_ gases was leaked into the inductively coupled plasma chamber at constant flow ratios of 15, 10, and 30 sccm. The plasma discharge was generated at 400 W where an additional 100 W was used for biasing to accelerate and direct the ions to the surface. In this way, the polymeric NWs with an aspect ratio of up to 700 were produced on the surface of a large number of polymers including PET, Kapton, Dura film, PS, and polydimethyl siloxane (PDMS). The length of the NWs exhibits a linear trend with the exposure time, whereas the diameter remains constant at a value around 100 nm for a given thickness of the mask on the surface. On the other hand, the density of the NWs is strongly influenced by the thickness of the coated metal. The scanning electron microscopy (SEM) images of NW with respect to various thicknesses of the mask and the relationship of the etching rate with the plasma exposure time are presented in Figure 8.

**Figure 9:**
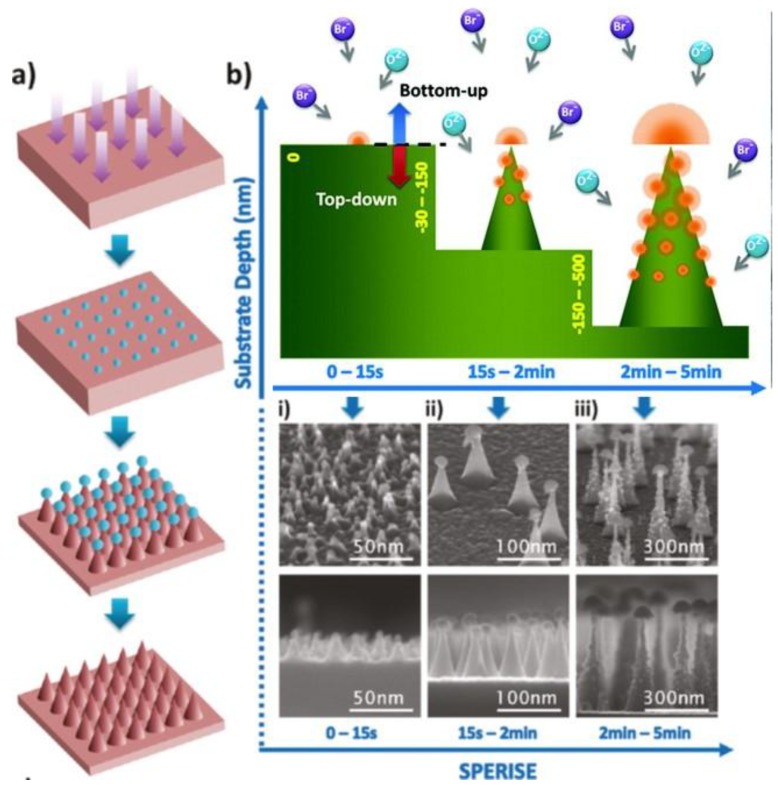
Simultaneous plasma enhanced reactive ion synthesis and etching (SPERISE) process and Si nanocone formation mechanism. (**a**) Process flow of the nanomanufacturing process: Pseudo randomly distributed silicon oxybromide nanodots are synthesized on the planar silicon substrate surface in the first few seconds of the SPERISE process. The oxide nanodots grow to hemispheres by a phase-transition nucleation process and act as a protective nanomask for the simultaneous reactive ion etching of the silicon underneath. Depending on the growth rate of the oxide hemispheres and the crystalline structures of the silicon substrates, nanocones with different aspect ratios are formed. The silicon oxybromide nanohemispheres on top of the nanocones are removed by wet etching; (**b**) Detailed schematic drawing of the three typical stages in the SPERISE process: Bromine and oxygen reactive ions interact with silicon to form synthesized oxide hemisphere and dots (orange) and etched silicon cone structure (green). Both the illustrations and corresponding SEM images at (i) 0–15 s; (ii) 15 s–2 min; and (iii) 2–5 min in the SPERISE process manifest this unique nanomanufacturing method (Reproduced with permission from [129]. Copyright American Chemical Society, 2011).

In many of the presented examples, the surface nanostructuring is achieved by using an appropriate mask/template. Nevertheless, it was not necessary to introduce a mask prior to processing. A typical example is found where simultaneous plasma-enhanced reactive ion synthesis and etching (SPERISE) is employed. This technique was first used during the treatment of a Si wafer inside HBr-O_2_ gas plasma (Figure 9a). In the primary step, the halogen species interact with the surface, and Si atoms are released into the vapor phase. This excited Si is then combined with the O and Br reactive species to form an etch-resistant silicon oxy bromide complex, which is deposited back on the surface. These deposits then act as masks and support the non-uniform etching on the surface. As a result of this processing, the mushroom-like Si NWs are formed, where an etch mask is left on the top (Figure 9b). The removal of the etch mask is done later with relatively simple mild chemical treatment [129].

Another application of plasma selective etching includes the study of filler dispersion inside the composite matrix. The filler dispersion inside the polymer matrix is the deciding factor for its bulk properties including ductility, hardness, impact resistance, *etc*. Conventional methods such as cryogenic breaking are generally used to monitor the dispersion of the fillers [130,131]. However, this method lacks precision for the determination of dispersion due to the possible displacement of the fillers during the distortion of the matrix. To overcome this, dissociated plasma etching is applied where the surface polymer layer is selectively removed to expose the embedded fillers. A fast and selective removal of the surface polymer from the composite materials including powder coatings and paint films with the dissociated oxygen plasma followed by SEM imaging enables the monitoring of the filler dispersion [132,133,134,135]. The same strategy allowed for the optimization of the bonding process efficiency of powder coatings with other materials, where bonding is enabled through disclosed but still embedded fillers. Due to the inhomogeneity in the plasma etching of various components inside the composite material, the resulting surface acquires relatively high surface roughness due to the exposed fillers. Along with suitable plasma functionalities, increased surface roughness is gained, which additionally improves the metallization of the surface [136,137]. Similarly, the fabrication of counter electrodes for solar cell application was done from a PP/CNT nanocomposite, where O_2_ plasma reactive ion etching was used to remove the thick protruding layer of PP on the surface to expose the embedded CNTs. As a result, the charge transfer resistance of the composite surface is diminished to much lower values [138].

The applications of plasma selective etching are further extended for achieving improved bio- and chemo-sensing properties of semiconducting materials. As shown in one recent report, the gas-sensing properties of a composite membrane prepared by electrospinning process from the aq. solution of polyvilnyl alcohol (PVA) and SnO_2_ was improved by O_2_ plasma treatment. The resulting SnO_2_ material showed a notable sensing response towards very low concentrations of ethanol vapor (~1 ppb). The enhanced sensitivity was featured because of the high specific surface area of the ripple-like structures obtained after plasma treatment followed by annealing at 500 °C [139]. However, the metal oxide nanodevices are less preferred due to their inability to operate at room temperature for sensing applications that could be replaced by conducting polymers, carbon allotropes or their suitable composites [140,141,142]. The sensing ability of such materials, especially in the context polymeric composites are further improved by plasma modification of the surface. As demonstrated by Raghu and co-workers, plasma modifications of the MWCNT composite with conducting polymers indicated greater effects on sensitivity and selectivity towards various volatile organic compounds [143]. However, a better understanding of how plasma improves the sensitivity and selectivity is still to be studied extensively for improving the sensor properties. While in the case of bio-sensors, the sensitivity is found to be controlled by the covalent attachment of the bio-molecule to the sensing material. For this application, the removal of the polymer matrix and the functionalization of the exposed MWCNTs on the surface of a pristine PS/MWCNT composite yielded an opportunity to covalently attach antibodies and to fabricate advanced immunosensors [144].

**Figure 10:**
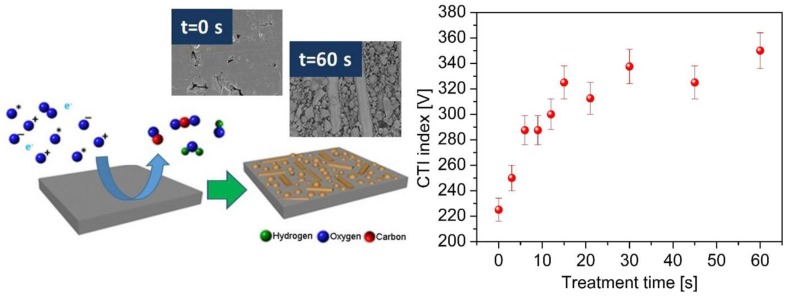
Plasma surface interaction of the glass-filled composite with corresponding SEM images for non-treated and plasma-treated samples for 60 s. The graph represents the variation of comparative tracking index (CTI) performance with plasma exposure time (Reproduced with permission from [101]. Copyright Royal Society of Chemistry, 2015, Year.”).

One of the most recent applications of plasma chemical etching was the improvement of the insulation properties of polymeric composites. The insulating properties of various polymeric materials are standardized in terms of comparative tracking index (CTI) as per international electrotechnical commission (IEC) grading. The origin of poor insulating properties of composite materials is an aftereffect of the polymer charring on the surface at a high voltage electric arc. This issue is commonly tackled by adding suitable fillers, which forms little or no char on the surface [145]. However, this is inadequate due to the thin polymer layer on top of the embedded fillers. The simplest solution to overcome this situation is the selective removal of the surface polymer layer by an optimized plasma discharge. The cold O_2_ plasma removal of the surface polymer from the glass embedded phenolic resin composite was demonstrated to improve CTI performances up to 56% (Figure 10) [101]. The performance level increased with the decrease in the surface polymer content along the prolonged plasma treatment times.

## 6. Conclusions and Research Challenges to Tackle

This review attempted to outline the interdisciplinary applications of plasma etching and the selective etching of polymer-based materials from different branches of science. Moreover, it looks at the origin of selectivity and attempts to find answers bringing together an understanding of plasma properties with very diverse results of plasma–surface interactions through the note to nanomaterials. Among various plasma modifications, neutral dense and ion free cold plasmas are have been found to be efficient, especially for treating delicate polymeric and biomaterials to avoid unwanted surface damages and thermal effects. The plasma-induced functionalization and etching of polymer substrates is preferred to wet chemical etching and UV irradiation for designing the surface chemistry, surface morphology, and surface energy. These plasma induced modifications enable the attachment of various materials and biomolecules onto the surface. Additionally, plasma-induced hydrophobization of the surface can effectively increase the water-resistant behavior of the surface to improve anti-aging, anti-fouling, and corrosion-resistant properties. Such improvements in the surface properties are directly connected to structural and morphological changes.

The high-energy particles inside the plasma are able to distinguish various bond types under controlled process parameters. Utilizing the difference in the chemical stability of various bond types on the same molecule, delicate materials such as GNRs have been fabricated. The comparison of etching rate for different classes of polymeric materials has revealed that the difference in the etch rate is connected with both physical and chemical properties of the material. The presence of aromatic moieties has reduced the etching rate by radical quenching, whereas the surface cross-linking has reduced the etching rate for branched polymers. Generally, the etch rates are lower for hydrocarbon polymers compared to functionalized macromolecules. Additionally, a higher extension of crystallinity has reduced the plasma etch rates for various polymers, presented through reports on a few examples, namely, PET, LDPE, and HDPE. Thus, existing differences in the etching rates for various polymeric substrates have been employed in the surface structuring of block copolymers in applications, especially for the semiconductor industry. The pronounced applications of plasma selective etching of composite materials have been found to be efficient for reducing the dimensionalities of materials such as CNT or graphene sheets for advanced applications in electronics, electrical, and sensing devises. Furthermore, the preferential etching of composite surfaces has been effectively used for a simple and low-cost synthesis of polymer NWs with a controlled aspect ratio.

The plasma etching rate and etching selectivity of micro/macro molecules are well connected with the strength of the available bonds in the material. However, the side reactions such as radical quenching and surface cross-linking significantly affect the etching rate. Thus, the optimization of the process parameters is essential for achieving etching selectivity in different systems. Plasma is used as a single operating tool in only one/a few step(s) throughout multistep processes for demanding applications. More frequently used plasma has been found as one of the steps in multi-processing. This is clearly visible in many of the discussed examples, including the synthesis of GNR, graphene nanomesh, patterning the surface of block copolymers, *etc*. In some of the presented examples, the involved wet chemistry had slight adverse effects on the properties of the final product. An additional challenge in front of the plasma community is the pinpoint control of various plasma species and their reactions on the atomic or molecular level. Such developments are important, especially for such remarkable applications as plasma nanoscience and plasma medicine.

## Figures and Tables

**Table 1:** Comparison of wet chemical etching *versus* plasma etching.

	Wet Chemical Etching	Plasma Etching
*Etchant:*	Chemical (acids, alkali, *etc.*).	Reactive gas (radicals, ions, *etc.*).
*Etch rate and selectivity:*	High.	Good, controllable.
*Advantageous:*	Low equipment cost, fast processing and easy to implement.	Capable of small scale etching (~10 nm), no contamination issues, no hazardous chemicals, ecologically benign technology.
*Disadvantageous:*	Inadequate to define small feature size less than 1 µm, handling of hazardous chemicals, contamination issues, ecologically unfriendly technology with need of waste processing.	High equipment cost, implementation dependent on application, potential radiation damage.
*Directionality:*	Only isotropic etching.	Can be isotropic or anisotropic.

**Table 2:** Relative rates of O_2_ plasma removal (k_rel_) and G_s_-values for selected polymers (Reproduced with permission from [72]. Copyright John Wiley and Sons, 2004).

No.	Polymer	k_rel_	G_s_
1	Poly(α-methylstyrene)	1.11	0.3
2	Polyphenyl methacrylate	1.33	-----
3	Polyviny1 methyl ketone	1.48	-----
4	Polymethy1 methacrylate (PMMA)	2.37	1.2
5	Polymethyl methacrylate-co-methacrylonitrile (94:6 mol %)	2.70	2.03
6	Polyisobutylene	3.56	4
7	Polybutene-1 sulfone	7.11	8
